# Host protein cleavage by Dengue and Zika virus NS3 proteases: from substrate identification to potential biological consequences

**DOI:** 10.3389/fcimb.2026.1899950

**Published:** 2026-07-09

**Authors:** Umar Saidu, Bbumba Patrick, Wen-Chi Su

**Affiliations:** 1 Graduate Institute of Biomedical Sciences, China Medical University, Taichung, Taiwan; 2 Department of Biochemistry, Ahmadu Bello University, Zaria, Nigeria; 3 International Master’s Program of Biomedical Sciences, China Medical University, Taichung, Taiwan; 4 Department of Medical Research, China Medical University Hospital, Taichung, Taiwan; 5 Drug Development Center, China Medical University, Taichung, Taiwan

**Keywords:** Dengue virus, host protein cleavage, immune evasion, NS2B-NS3 protease, NS3 protease, viral pathogenesis, Zika virus

## Abstract

Dengue virus (DENV) and Zika virus (ZIKV) are medically important orthoflaviviruses that utilize the multifunctional NS3 protease, in complex with its cofactor NS2B, for viral replication and host modulation. Here, we summarize current knowledge of host proteins targeted by NS3 proteases and discuss recent advances in proteomic and computational approaches for identifying these substrates. We further discuss evidence showing that NS2B3-mediated cleavage alters innate immune signaling, autophagy, protein translation, and cytoskeletal dynamics. In addition, we compare the host substrate specificities of DENV and ZIKV proteases, emphasizing both shared mechanisms and virus-specific differences that may contribute to their distinct disease manifestations. A deeper understanding of NS3-mediated host protein cleavage will provide critical insights into orthoflavivirus biology and further establish NS3 as a promising target for antiviral intervention.

## 1 Introduction

Orthoflaviviruses are enveloped, positive-sense, single-stranded RNA viruses maintained in zoonotic cycles by infected mosquitoes and ticks (Lindqvist et al., 2018). Their bites transmit pathogens, including Zika virus (ZIKV), Japanese encephalitis virus (JEV), West Nile virus (WNV), dengue virus (DENV), yellow fever virus (YFV), and tick-borne encephalitis virus (TBEV). These infections give rise to a broad spectrum of diseases, encompassing systemic hemorrhagic conditions and neurological disorders (Xiong et al., 2025). DENV remains a major global public-health threat (WHO, 2025), whereas ZIKV has drawn particular attention because of its neurotropism and congenital disease associations.

The orthoflavivirus genome encodes a single polyprotein that is cleaved into three structural (C, prM, E) and seven non-structural (NS1, NS2A, NS2B, NS3, NS4A, NS4B, NS5) proteins, which primarily mediate viral replication and assembly (Sousa et al., 2024). NS3 contains an N-terminal serine protease that processes the viral polyprotein and a C-terminal NTPase-driven RNA helicase required for genome replication and RNA synthesis. For overall clarity, the term “NS3” refers to the full-length viral protein, “NS3pro” to the isolated N-terminal protease domain, and “NS2B3” to the catalytically competent NS2B-NS3 protease complex.

**Figure 1:**
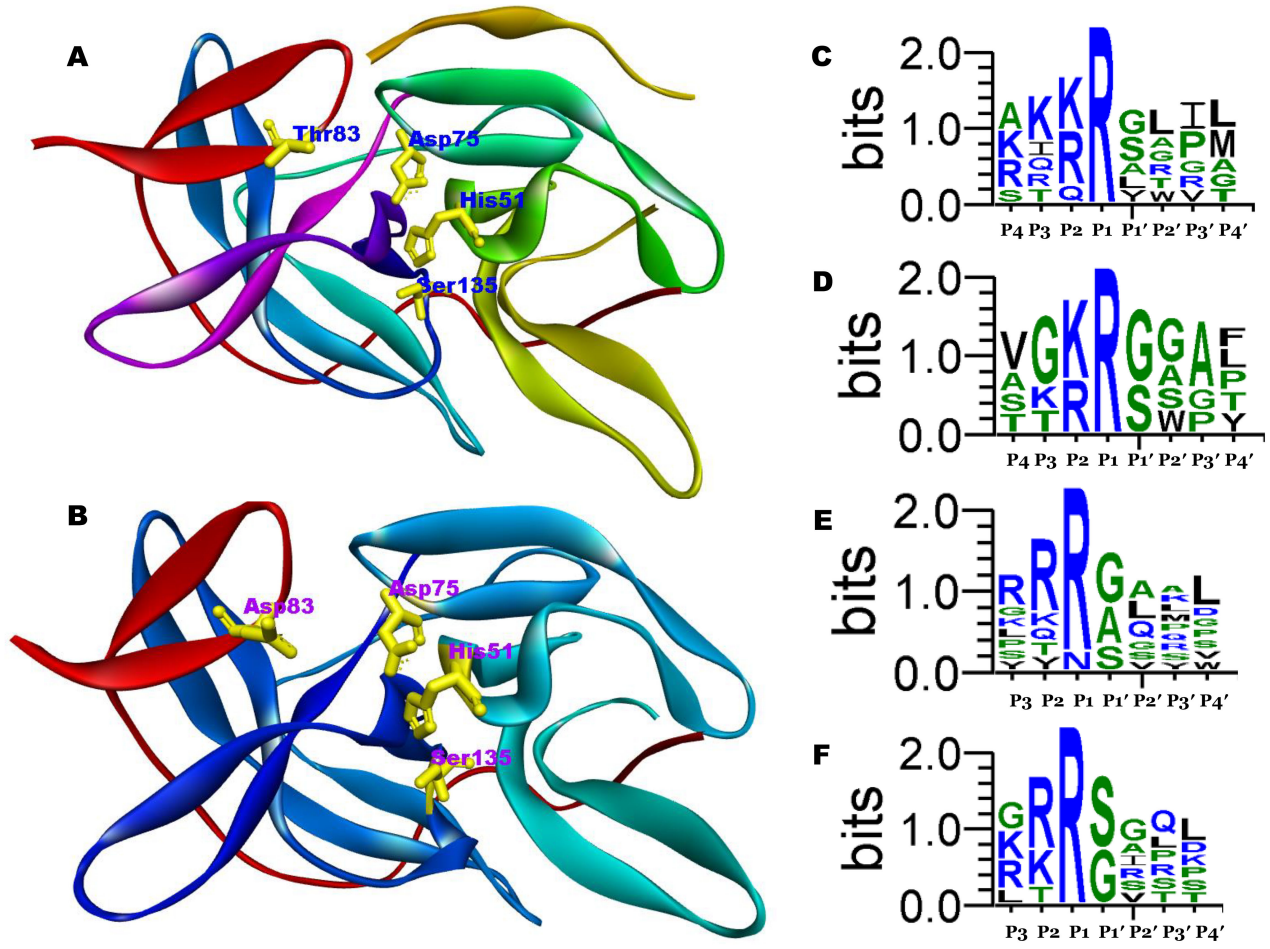
Structural features and substrate specificity of DENV and ZIKV NS2B3 proteases. **(A, B)** show DENV and ZIKV NS2B3 protease complex, respectively, including the NS2B cofactor (red), and the catalytic triad residues (His51-Asp75-Ser135) within the active site. **(C–F)** The cleavage site motif recognized by NS2B3 proteases is presented using the P4-P4′ nomenclature. The sequence logos were generated from known viral polyprotein [**(C)** DENV and **(D)** ZIKV], and host-substrate [**(E)** DENV and **(F)** ZIKV] cleavage sites using the WebLogo web server. The P1 position is predominantly occupied by Arg, while small residues such as Gly, Ser, or Ala are favored at the P1′ position, reflecting substrate specificity of DENV and ZIKV NS2B3.

The catalytic triad of DENV and ZIKV NS3 is His51-Asp75-Ser135, positioned in the NS2B-NS3 protease active site (**Figures 1A, B**) (Noble et al., 2012; Dang et al., 2022). NS2B cofactor activity is primarily mediated by a central hydrophilic region comprising approximately 35% of the full-length protein, indicating that the entire NS2B sequence is not required for protease function (Falgout et al., 1991; Erbel et al., 2006). Despite sharing similar functions, the NS2B cofactor regions of DENV and ZIKV exhibit only 41% sequence identity and differ at residue 83, where DENV contains Ser/Thr, and ZIKV contains Asp (Hill et al., 2018). This variation contributes to differences in substrate recognition, particularly at the P2 position. Analysis of cleavage site sequences from viral polyproteins and host substrates revealed consensus patterns, which are illustrated as sequence logos in **Figures 1C–F**. The NS2B3 protease complex cleaves substrates predominantly at sites containing basic residues at P2 and P1, often Lys/Arg combinations such as KR, RR, RK, or QR, followed at the P1’ position by a small residue such as glycine, alanine, or serine (**Figures 1C–F**) (Lin et al., 2014). In fact, both processes are crucial for establishing a successful infection cycle and pathogenesis (Wahaab et al., 2021). Given that ZIKV NS3 shares the same conserved catalytic triad as DENV, which causes the largest global disease burden, we review herein the experimentally validated host substrates of DENV and ZIKV NS2B3 proteases and discuss their biological significance in virus–host interactions.

## 2 Approaches for identifying host protein cleavage by orthoflavivirus NS3 proteases

Researchers use integrated proteomic and computational approaches to identify host substrates cleaved by orthoflaviviral proteases. The following section provides a brief summary and evaluation of commonly used techniques.

### 2.1 Proteomic approaches to identify host protein cleavage by DENV and ZIKV NS2B3 protease

Mass spectrometry-based proteomics enables unbiased discovery of host protein cleavage events. Proteomics-based identification has transitioned from individual candidate assays to comprehensive screens that utilize the chemical signature produced by peptide-bond hydrolysis (Hill et al., 2018; Riedl et al., 2019; Dabrowska et al., 2021). Among them, N-terminomics strategies represent the most widely used mass spectrometry approaches for protease substrate profiling, with methods such as terminal amine isotopic labeling of substrates (TAILS) and subtiligase-based enrichment allowing direct identification of neo-N-terminal peptides generated upon proteolysis (Mintoo et al., 2021). In TAILS, isobaric tags such as TMT or iTRAQ are used to block native and neo-N-terminal peptides, followed by trypsin digestion and selective depletion of internal tryptic peptides. The enriched N-terminal peptides are then analyzed by LC-MS/MS to determine substrate identity and cleavage sites (Mintoo et al., 2021). This strategy has identified multiple human substrates of Dengue and Zika virus NS2B3 proteases (Hill et al., 2018; Mintoo et al., 2021; Bhattacharya et al., 2023; Gandhi et al., 2023), including cyclic GMP-AMP synthase (cGAS), autophagy-related protein 16-1 (ATG16L1), and eukaryotic translation initiation factor 4 gamma 1 (eIF4G1).

Subtiligase-based enzymatic labeling biotinylates free protein N-termini, enabling streptavidin -mediated enrichment and LC-MS/MS identification of proteolytic cleavage events (Weeks and Wells, 2020). These approaches directly reveal P1-P1’ cleavage-site specificity, which is important for understanding NS3 substrate recognition patterns (**Figures 1C–F**) (Shiryaev et al., 2007). Despite the important role of N-terminomics in accelerating the discovery of novel substrates, several limitations should be considered. For example, low-abundance proteins may be missed, and secondary proteolytic events can generate false-positive signals by producing cleavage products unrelated to direct protease activity. In addition, protease expression systems do not fully recapitulate the complexity of proteolytic events that occur during viral infection. Consequently, mass spectrometry-based approaches typically generate a list of candidate substrates, which must be further validated through biochemical cleavage assays, mutational analyses of cleavage motifs, and/or infection-based experiments.

### 2.2 Computational methods for identifying host protein cleavage by DENV and ZIKV NS2B3 protease

Computational approaches have become valuable complements to experimental studies for identifying candidate host substrates of orthoflavivirus NS2B3 proteases and for systematically evaluating determinants of substrate specificity. Methods such as sequence-based prediction, structural modeling, machine learning, and network analysis are increasingly used to identify potential enzyme substrates. In silico sequence motif analysis and homology profiling have been successfully used to identify crucial host targets, including STING (stimulator of interferon genes), which is cleaved by dengue and Zika virus proteases to evade antiviral responses, and EDRF1, which is important for platelet development (Aguirre et al., 2012; Ding et al., 2018; Gandhi et al., 2023).

A key advance in this area is the incorporation of machine-learning classifiers that integrate biochemical features with structural accessibility predictions. Stanley and coworkers developed a two-stage model to identify protein sequences that resemble known viral cleavage sites and to assess whether these sites are located in flexible, exposed regions of the protein where cleavage could occur. This is designed to identify putative host protein targets of DENV protease, which revealed a total of 3,991 putative cleavage sites, narrowed to 257 high-confidence candidate sites using machine-learning predictions (Stanley et al., 2020). However, despite the growing use of machine learning and structure-guided computational approaches in virology, their application to identifying host cellular protein targets of DENV and ZIKV NS2B3 proteases remains relatively limited.

### 2.3 Complementary strategies for identifying host protein cleavage by DENV and ZIKV NS2B3 protease

The identification of NS2B3 host substrates will benefit from diverse strategies, including proteomics, computational methods, and experimental techniques. Candidate-based biochemical cleavage assays are an established method to identify direct proteolysis. While peptide libraries are excellent for determining protease substrate specificity and defining cleavage patterns, cleavage reporter assays are a straightforward way to evaluate protease activity in living cells (Gruba et al., 2016; Pahmeier et al., 2021). The reporter cleavage has been demonstrated in DENV, which takes advantage of NS2B3-specific cleavage sequences inserted into the reporter construct. The virus-encoded proteins expressed in the cells can be cleaved, and the signal can be quantitatively analyzed (Pahmeier et al., 2021). Techniques like hybrid combinatorial substrate library, a peptide-library substrate profiling, use substrate specificity profile of NS2B3 protease to obtain the protein sequences that are recognized by the NS2B3, synthesize selective substrate and activity-based probe for screening potential substrates of the orthoflaviviral NS2B3 protease (Rut et al., 2020). This may provide the starting point for identifying cellular substrates of DENV and ZIKV NS3 proteases, paving the way for designing antiviral compounds. NS2B3 substrate candidates may derive from protein interaction and proximity labeling methods, but these methods only demonstrate protease-substrate interaction. Because virus-host interaction is dynamic, an integrative approach may be required to fully understand protein-protein interaction and identify potential subcellular substrates during DENV and ZIKV infection (Song et al., 2021). For NS2B3 substrate validation, additional assays, including but not limited to the use of inactive protease variants, cleavage-site mutation, infection-based assays, and protease inhibitors, are required.

## 3 Host cellular substrates of DENV and ZIKV NS2B3 protease and the potential biological consequences of their cleavage

The viral NS2B3 protease acts as a multifunctional virulence factor by targeting host cellular substrates (**Table 1**) and disrupting key cellular systems, thereby creating an environment permissive for efficient viral replication and pathogenesis. Its activity affects multiple interconnected biological processes, such as innate immune signaling (Yu et al., 2012; Chen et al., 2017), autophagy (Zaffagnini and Martens, 2016; Zhou et al., 2024; Ahmed et al., 2025; Freppel et al., 2025), protein translation (Hill et al., 2018; Lan et al., 2023; Zhou et al., 2024), endoplasmic reticulum (ER) homeostasis (Lennemann and Coyne, 2017; Hill et al., 2018), cytoskeletal organization (Li et al., 2019a; Liu et al., 2021), and metabolism (Ogire et al., 2024; Ahmed et al., 2025). Cleavage of host substrates can change protein stability, impair antiviral signaling pathways, and reprogram metabolic fluxes and cellular trafficking to support viral propagation. Because of the protease’s precise substrate specificity and the need to maintain host cell viability during infection, NS3-mediated cleavage often results in selective modulation rather than a complete shutdown of host pathways.

**Table 1:** Host substrates targeted by DENV and ZIKV NS3 proteases and functional consequences.

Functional grouping	Host substrate	Virus	Biochemical cleavage	Cleavage validation	Cleavage site mapped	Cellular pathway affected	Potential consequence of protease-mediated cleavage	References
Disruption of host immune signaling and mitochondrial function	MITA (STING)	DENV ZIKV	Yes Yes	Cell infection Cell and mice infection	LRR↓G^97^and SRY↓RG^79^ SRY↓RG^79^	Type I interferon signaling cGAS-STING signaling	Impaired MITA function, inhibition of IRF3 activation and IFN production, promoting immune evasion Evasion of cGAS/STING- dependent signaling	(Yu et al., 2012; Stabell et al., 2018) (Ding et al., 2018)
	cGAS	DENV	Yes	*in vitro* cleavage assay using recombinant proteins	^123^QR↓GA^126^ and ^61^KK↓SA^64^	cGAS-STING signaling	Reduced DNA binding and enzymatic activity	(Bhattacharya et al., 2023)
	Autophagy-related protein 16-1 (ATG16L1)	DENV ZIKV	Yes Yes	*in vitro* cleavage with cell lysates Cell infection and *in vitro* cleavage with cell lysates	FGRR↓SV^288^	Interferon signaling	Impaired antiviral response	(Hill et al., 2018)
	IκBα IκBβ	DENV DENV	Yes Yes	Cell infection Cell infection	*PR↓GS^64^ *ERR↓G^128^	NF-κB signaling	Constitutive NF-κB activation, excessive inflammation, and induction of host cell death	(Lin et al., 2014)
	JIP4	ZIKV	Yes	*in vitro* cleavage with cell lysates	VKRR↓SS^594^	IFN-α antiviral response	Destabilizes JNK pathway scaffolding, leading to reduced activation of downstream transcription factors involved in antiviral and stress responses	(Hill et al., 2018)
	TAK1 (MAP3K7)	ZIKV	Yes	*in vitro* cleavage with cell lysates	PRRR↓SI^440^	MAPK signaling	Disruption of TAK1 signaling impairs stress-activated MAPK pathways, weakening cellular responses to viral infection and inflammatory stimuli	(Hill et al., 2018)
	MFN1 MFN2	DENV DENV	Yes Yes	Cell infection Cell infection	PRN↓A^541^ SRR↓A^565^	Mitochondrial dynamics	Impaired mitochondrial fusion, weakened antiviral signaling, and enhanced cytopathicity	(Yu et al., 2015)
	GrpEL1	DENV	Yes	Cell transfection/infection	KR↓A^82^ QR↓S^93^	Mitochondrial protein folding	Mitochondrial dysfunction due to loss of mtHsp70 co-chaperone activity	(Gandikota et al., 2020)
Autophagy manipulation	Sequestosome 1 (SQSTM1/p62)	ZIKV	Yes	Cell culture transfection/infection	GGKR↓SR^267^	Autophagy	Defective cellular anti-NS3 response due to non-sequestration of NS3 and lysosomal degradation	(Zhou et al., 2024)
	FAM134B	DENV, ZIKV	Yes	Cell culture transfection	RTR↓G^143^	Selective autophagy (reticulophagy)	Suppression of ER turnover, preservation of ER membranes, and enhanced replication organelle formation	(Lennemann and Coyne, 2017)
Host Translation	Eukaryotic translation initiation factor 4 gamma 1 (eIF4G1)	DENV ZIKV	Yes Yes	*in vitro* cleavage with cell lysates Cell infection and cell lysates	DKRR↓GG^1034^	Host cap-dependent translation initiation	Reducing translation of host mRNAs, including those encoding antiviral and stress-response proteins.	(Hill et al., 2018)
Cell cycle, cytoskeleton, and development	Septin-2	ZIKV	Yes	Cell culture expression/transfection	RLKR↓GGR^309^	cytokinesis and cytoskeletal organization	Failed cytokinesis, reduced cell proliferation and survival,	(Li et al., 2019a)
	Human Kinesin-5 (Kif11/HsEg5)	ZIKV	Yes	Cell culture transfection	GAAKR↓T^222^ PRNKR↓G^193^ AVDKR↓A^283^	Cell cycle/mitotic spindle assembly pathway	cell cycle abnormalities and potential mitotic defects	(Liu et al., 2021)

*cleavage site was estimated based molecular weight of cleavage fragments.

Importantly, not every phenotype discussed below is interpreted as a direct consequence of cleavage alone; where the causal linkage remains incomplete, we describe the phenotype as associated with NS3/NS2B3 activity or as supporting a mechanistic model rather than demonstrating *in vivo* causality. Beyond its proteolytic activity, NS3 interacts with host proteins to modulate diverse cellular processes. Moreover, several studies have reported phenotypes attributed to NS3, although the causality in some cases may be indirect. In this review, we primarily focus on host proteins experimentally validated as substrates of NS3-mediated proteolytic cleavage and outline the cleavage site and potential biological consequences of cleavage in **Table 1**.

To distinguish experimentally validated cleavage events from broader NS3-associated phenotypes, host factors discussed in this review are categorized according to the strength of supporting evidence. Validated cleavage substrates are defined as proteins for which NS3/NS2B3-dependent proteolysis and a cleavage site have been experimentally demonstrated. Putative cleavage targets/protease-sensitive factors are proteins whose cleavage or degradation has been reported, but for which cleavage sites remain incompletely characterized or direct proteolysis has not been conclusively established. NS3-associated pathway perturbations are cellular processes or proteins whose altered activity has been linked to NS3/NS2B3 expression or infection, but for which direct protease-mediated cleavage has not been demonstrated.

### 3.1 Protease-mediated disruption of host innate immune signaling and mitochondrial function by NS3/BS2B3

DENV and ZIKV evade innate immunity through NS2B3-mediated cleavage of key signaling adaptors in the type I interferon (IFN-I) pathway (Aguirre et al., 2012; Ding et al., 2018; Bhattacharya et al., 2023). A central target is MITA (also known as STING), which acts as an antiviral restriction factor. DENV NS2B3 physically interacts with and cleaves human MITA at a defined site (LRR↓G^97^), but not its murine homolog MPYS. This cleavage suppresses IRF3 activation and subsequent type I interferon (IFN-I) induction in response to viral infection or nucleic acid sensing (Yu et al., 2012). In a different study, the cleavage site was identified as within (SRY↓RG^79^) (34), which is very similar to the ZIKV cleavage site (SRY↓RG^79^) (**Table 1**), confirming it as the primary target of NS2B3 protease (Ding et al., 2018). The discrepancies observed from these studies likely arise due to strain-dependent differences, alternative cleavage site or interpretation of cleavage products, which remained for further experimental revalidations. The human STING signaling is disrupted upon cleavage (Yu et al., 2012), thereby preventing the induction of IFN-β and viperin in dendritic cells. Consistently, inefficient DENV replication has been observed in mice carrying the alteration in the STING LRRG motif (Chen et al., 2017; He et al., 2025). On the other hand, ZIKV NS3 and NS2B3 target mitochondrial antiviral signaling protein (MAVS) and MITA, respectively (Li et al., 2019b), leading to downregulated IFN-I production and promoting viral persistence and pathogenesis. Although cleavage and protease interaction with MITA/STING have been experimentally demonstrated through cellular infection-based validations (**Table 1**), the precise cleavage site in MAVS has not yet been fully mapped, and whether MAVS reduction occurs exclusively through direct proteolysis or additionally involves host degradation pathways remains unclear.

Cyclic GMP-AMP synthase (cGAS) was shown to be efficiently cleaved through *in vitro* biochemical assay by the DENV NS2B3 protease at major ^123^QR↓GA^126^ and minor ^61^KK↓SA^64^ cleavage sites (Bhattacharya et al., 2023). DENV and ZIKV infection also alter the levels of ATG16L1, which is important in antiviral activity against murine norovirus (Hwang et al., 2012; Hill et al., 2018). Cell and infection-based validations demonstrated that DENV and ZIKV use NS2B3 to cleave ATG16L1 at FGRR↓SV^288^, thereby impairing the host antiviral responses (**Table 1**).

DENV NS2B3 protease also targets host NF-κB inhibitory proteins by cleaving IκBα (PR↓GS^64^) and IκBβ (ERR↓G^128^), generating 30- and 33-kDa fragments. Although these cleavage sites were predicted based on size using cell-based validation, DENV protease potently activates NF-κB signaling, leading to dysregulated inflammatory responses and induction of host cell death, thereby contributing to pathogenesis (Lin et al., 2014). Additionally, ZIKV NS2B3 cleaves C-Jun amino-terminal kinase interacting protein 4 (JIP4), a protein involved in IFN-α-mediated antiviral responses at VKRR↓SS^594^ and mitogen-activated protein kinase kinase kinase 7 (TAK1/MAP3K7) at PRRR↓SI^440^ in cell lysates (**Table 1**) (Hill et al., 2018). DENV NS3 also cleaves human IgG and DENV-neutralizing antibodies, generating λ free light chains (FLCs), as demonstrated by a cell-free *in vitro* cleavage assay; however, the cleavage site has not yet been mapped (Wang et al., 2023).

DENV NS2B3 targets mitochondrial dynamics by cleaving mitofusin-1 and 2 (MFN1/2). MFN1 is required for efficient RIG-I-like receptor signaling and IFN-I production, while MFN2 maintains mitochondrial membrane potential to protect cells from death (Lee et al., 2017). Evidence from biochemical and cell infection shows that DENV NS2B3 cleaves MFN1 at ^538^PRN↓A^541^ and MFN2 at ^562^SRR↓A^565^ in a protease-dependent and caspase-independent manner, disrupting mitofusin function and mitochondrial fusion (Yu et al., 2015).

DENV NS3 also targets proteins in the mitochondrial matrix, where the NS3pro and the NS3 protease-helicase fragment cleave GrpEL1, a critical cochaperone of mitochondrial Hsp70, at (KR↓A^82^ and QR↓S^93^), identified through biochemical and transfection/infection-based validations (**Table 1**) (Gandikota et al., 2020). Because these experiments rely substantially on defined constructs, the distinction between full-length NS3, NS3pro, and NS2B3 is important when extrapolating these findings to infection-context biology.

Furthermore, DENV employs NS3 and NS2B3 to target different cellular compartments and may collectively affect platelet development (Gandhi et al., 2023). In the nucleus, NS2B3 cleaves the transcription factor EDRF1, disrupting the EDRF1-GATA1-spectrin axis that is essential for platelet biogenesis. This phenomenon was observed through biochemical and co-transfection systems without the cleavage site experimentally validated. In parallel, mitochondrial-localized NS3 cleaves GrpEL1, leading to mitochondrial dysfunction and may potentially contribute to platelet abnormalities (Gandhi et al., 2023).

### 3.2 Targeting of autophagy pathways and remodeling of endoplasmic reticulum stress responses by ZIKV and DENV NS2B3 protease

Selective autophagy is a highly organized homeostatic process that enables cells to remove damaged organelles, misfolded proteins, and invading pathogens through lysosomal degradation (Zaffagnini and Martens, 2016; Vargas et al., 2023). NS2B3 suppression of autophagy is primarily through direct cleavage of host autophagy machinery rather than by promoting proteasomal or lysosomal degradation of the receptors themselves. For example, endoplasmic reticulon-phagy receptors are susceptible to NS2B3-mediated processing (Lennemann and Coyne, 2017). ZIKV NS2B3 cleaves sequestosome 1 (SQSTM1/p62) at ^262^GGKR↓SRLT^269^ (Zhou et al., 2024). This was supported by biochemical, cell/infection-based experiments, providing clear evidence that SQSTM1/p62 is a bona fide substrate of the ZIKV NS2B3 protease. Normally, the human SQSTM1 function in the autophagic degradation of ZIKV NS3 and NS5 as part of the host antiviral response. Its cleavage generates truncated fragments that lack key UBA and PB1 domains, thereby impairing cargo recognition and oligomerization, promoting viral replication (Zhou et al., 2024). Another host antiviral factor, FAM134B-mediated reticulophagy, which restricts orthoflavivirus replication at early stages, is shown to be cleaved at a non-canonical site, RTR↓G^143^ as demonstrated through biochemical and transfection experiments (**Table 1**) (Lennemann and Coyne, 2017). Substitution of the predicted cleavage residue (R142A) abolished the observed activity. This cleavage disrupts FAM134B oligomerization and impairs reticulophagy-associated autophagosome formation, thereby preserving ER membranes for DENV and ZIKV replication (Lennemann and Coyne, 2017).

Recent findings revealed the involvement of endolysosomes in coordinating antiviral signaling, autophagy, membrane remodeling, and degradation of viral components (Post et al., 2025). In this wider context, the cleavage of SQSTM1/p62 and FAM134B potentially disrupts selective autophagy and interferes with endolysosomal framework involved in control of replication organelles and innate immune signaling. On the other hand, host cellular response mediates lysosomal degradation of NS3, highlighting the dynamic interplay between viral protease activity and host endolysosomal defense mechanisms.

### 3.3 Disruption of host translation and nucleocytoplasmic transport by NS2B3 protease

DENV and ZIKV NS2B3 proteases promote viral replication by selectively cleaving key components of the host translational and nucleocytoplasmic transport machinery (Lan et al., 2023; Zhou et al., 2024). The protease dismantles cap-dependent translation by cleaving human eIF4G1 at DKRR↓GG^1034^, as supported by biochemical and cell lysate assays (**Table 1**), resulting in functional depletion of the host translation apparatus, thereby favoring viral proliferation (Hill et al., 2018). The NS2B3 cleaves FG-nucleoporins of the nuclear pore complex (NPC), including Nup62, Nup98, Nup153, and TPR (translocated promoter region), thereby compromising NPC integrity (De Jesús-González et al., 2020). Although, the exact cleavage sites were not mapped, the phenomenon was shown through cell/infection-based experiments, which suggested degradation of FG-Nups during DENV and ZIKV infection and through expression systems. Pharmacological inhibition of serine proteases (leupeptin and TLCK) blocks Nup cleavage, while ectopic expression of catalytically active NS2B3, but not its inactive mutant, is sufficient to disrupt the nuclear pore ring, confirming a protease-dependent mechanism. NS2B3-mediated degradation of FG-Nups dismantles the NPC, perturbs nuclear-cytoplasmic trafficking, and facilitates the redistribution of viral and host factors (De Jesús-González et al., 2020; Liu et al., 2021).

### 3.4 Cytoskeletal reorganization and intracellular trafficking controlled by NS2B3

DENV and ZIKV modulate host cytoskeletal architecture, cell remodeling, and intracellular trafficking to promote viral replication and, in the case of ZIKV, may potentially contribute to neurotoxicity (Liang et al., 2016; Dar et al., 2017; Li et al., 2019a). DENV NS2B3 protease associates with nuclear receptor binding protein, a host factor involved in Golgi-ER trafficking, and interacts with Rac3, a member of the Rho-GTPase family (Chua et al., 2004). For these trafficking-associated host factors, interaction with NS2B3 has been reported; however, definitive cleavage sites and direct proteolytic processing have not yet been fully characterized. ZIKV infection may potentially be implicated in neurotoxicity through its effects on neural progenitor cells (Liang et al., 2016; Dar et al., 2017). ZIKV reduces cell growth and induces cell death by cleaving host proteins essential for neurogenesis via its NS2B3 protease (Li et al., 2019a). For instance, Septin-2, a cytoskeletal protein associated with neural cell division, promotes mitotic spindle organization and cytokinesis, and is cleaved at ^303^RLKR↓GGRK^310^ as experimentally validated through direct biochemical and cell culture transfection cleavage (**Table 1**). Similarly, human Kinesin-5 (Kif11/HsEg5), an essential homotetrameric motor protein required for mitotic spindle assembly and orientation, is directly targeted by ZIKV NS2B3. The protease cleaves multiple sites within the human kinesin-5 motor domain (^216^KGAAKR**↓**T^222^, ^187^DPRNKR**↓**G^193^, and ^276^GAVDKR**↓**A^283^) (Liu et al., 2021). This cleavage delays mitosis and hinders cell proliferation, which may contribute to impaired neurodevelopment and microcephaly associated with ZIKV infection.

### 3.5 Metabolic rewiring of infected cells through targeting of key enzymes

Several studies have demonstrated that DENV and ZIKV NS2B3 proteases alter host metabolic pathways, including lipid metabolism, oxidative stress responses, and autophagy-associated processes that facilitate viral replication. However, the precise cleavage site(s) and whether these effects arise from direct proteolysis or indirect metabolic remodeling remain unresolved. ZIKV NS3 mediates osteogenic responses and calcium deposition in the human brain. It does this by cleaving bone morphogenetic protein (BMP), a protein that promotes abnormal calcium deposition, into its active form, thereby committing it to osteogenic pathways (Chen et al., 2021). The NS2B3 protease affects lipid synthesis, glycolysis, and mitochondrial respiration (Ogire et al., 2024; Ahmed et al., 2025), thereby reshaping cellular energy utilization and biosynthetic capacity. DENV NS2B3 subverts host redox regulation by targeting the master antioxidant regulator Nrf2. NS2B3 promotes Nrf2 degradation through a proteasome-independent, lysosome-dependent mechanism, thereby suppressing Nrf2-driven antioxidant gene expression, leading to elevated reactive oxygen species (ROS), and promoting oxidative stress (Ferrari et al., 2020). DENV NS2B3 selectively targets mitochondrial complex I subunit and impairs the electron transport system to reprogram host cell metabolism (Sousa et al., 2024). Redox regulation is more than a consequence of viral infection and it is important to understand this in the context of antiviral immunity and flavivirus pathogenesis. Recent findings indicate redox regulation and its related metabolism of ROS, and endolysosomal signaling are tightly linked to antiviral responses. For example, plasmacytoid dendritic cells (pDCs) are the largest IFN-I producing cells and use a special redox metabolism to carry out their antiviral defense at the same time minimizing potential tissue and inflammatory damage (Post et al., 2025; Schloer et al., 2025; Rueschpler and Schloer, 2026). These findings maybe applicable to DENV and ZIKV infections since NS2B3 in DENV and ZIKV perturbs the redox balance in cells and regulates mitochondrial function. Increased ROS and reduced expression of Nrf2 are the result of DENV NS2B3-mediated Nrf2 cleavage.

Furthermore, aldolase A (ALDOA), a glycolytic enzyme, is downregulated at the protein level during both NS2B3 expression and ZIKV infection, implying that the virus reprograms cellular energy metabolism to favor its replication strategy (Tangsongcharoen et al., 2019). Evidences documented in these studies observed alterations in the identified host cellular substrates and have been demonstrated through *in vitro* cleavage, cell-based and infection-based experiments. Notably, although most of these metabolic targets should be viewed as NS2B3-associated pathway perturbations due to the lack of direct cleavage evidence, they still suggest potential biological impacts of NS2B3-mediated proteolysis.

## 4 Comparative analysis of host substrate specificity of DENV and ZIKV NS3 proteases

DENV and ZIKV encode highly conserved NS3 proteases that contain the catalytic triad His51, Asp75, and Ser135 and require the NS2B cofactor for full proteolytic activity (**Figures 1A, B**) (Hill et al., 2018; Shah et al., 2018; Riedl et al., 2019). Despite this conservation, their host substrate repertoires only partially overlap. DENV and ZIKV NS2B3 proteases inhibit innate immune signaling by cleaving adaptor proteins, such as MAVS and STING/MITA, thereby suppressing type I IFN responses. At the same time, some host proteins appear to be selectively cleaved by one virus but not the other, highlighting virus-specific substrate preferences (Yu et al., 2012; Hill et al., 2018). Available evidence suggests that these differences may reflect variations in NS2B3 conformational dynamics, cleavage-site accessibility, protease localization, and host protein abundance. Understanding these distinctions is important for elucidating orthoflavivirus pathogenesis and guiding the development of broad-spectrum antivirals.

The orthoflavivirus NS3 protease activity depends on the NS2B cofactor. *In vitro* studies using synthetic peptide and natural polyprotein substrates have shown that the NS2B cofactor is necessary for the catalytic activity of the NS3 protease (Falgout et al., 1991). Notably, ZIKV NS2B3 exhibits ~16-fold higher activity than the DENV protease toward the GRR-AMC substrate. Increased activity could indicate the need for rapid viral maturation for reproduction and evasion, or for rapid proteolysis to overcome the host cellular antiviral response. This increased activity was originally thought to be caused by residue Asp83 in NS2B (Lei et al., 2016; Hill et al., 2018). Although the Asp83 residue in the cofactor was previously proposed as a key determinant of the enhanced catalytic activity of the Zika virus NS2B3 protease, mutational analysis indicates that this residue alone is not sufficient to account for the observed activity differences. Substitution of Asp83 with asparagine resulted in only a modest (~2-fold) reduction in catalytic efficiency, and the mutant ZIKV protease retained higher activity than the corresponding WNV and DENV proteases. These findings suggest that increased Zika protease activity arises from cooperative effects of multiple residues or conformational features within the NS2B3 complex. Nonetheless, Asp83 may contribute to substrate recognition or specificity rather than serving as the sole driver of enhanced proteolytic activity.

Peptide-based studies show that ZIKV NS2B3 protease strongly favors a positively charged residue (Arg or Lys) at the P1 position (**Figure 1C**) (Gruba et al., 2016; Rut et al., 2017), while small residues, such as Gly or Ser, are favored at the P1′ position. This specificity profile closely resembles the documented ZIKV cleavage sites (Hill et al., 2018). In comparison, DENV NS2B3 protease exhibits a similar overall specificity, with a strong preference for dibasic residues at P1 and P2, enhanced cleavage by basic residues at P3/P4, and preferential accommodation of small residues at P1′ (especially Ser, followed by Gly or Ala), whereas P2′ is relatively unrestrained (Niyomrattanakit et al., 2006).

Differences in NS2B cofactors and their conformational changes could also be a possible explanation. DENV and ZIKV NS2B have around 41% sequence identity. Studies indicate that NS2B helps in the catalysis and binds to the substrate and stabilizes the protease in both its active and inactive forms (Grabski et al., 2025). It has been found that the ZIKV NS2B3 has a higher catalytic activity toward peptide substrates than its DENV counterpart. This could also mean that DENV and ZIKV can engage different substrates and thus pose different structural barriers to different host proteins, which explains why among many others JIP4, TAK1, SQSTM1/p62, Septin-2, and Kif11 (Hill et al., 2018; Li et al., 2019a; Liu et al., 2021; Zhou et al., 2024) have been found to be ZIKV substrates, and other proteins are more closely associated to DENV. Cellular localization can also determine which substrates are susceptible to proteolytic cleavage. NS3 and NS2B3 can be localized to multiple different cellular compartments such as the endoplasmic reticulum, mitochondria, the nucleus, as well as replication organelles. Mitochondrial NS3 in DENV has been shown to cleave GrpEL1 to disrupt the mitochondrial homeostasis while the NS2B3 in the nucleus can cleave EDRF1 (Gandikota et al., 2020; Gandhi et al., 2023). In the case of ZIKV NS2B3, the cleavage of mitosis and neurodevelopmentally associated substrates, like Septin-2 and Kif11, may reflect the protease’s access to its substrates within neural progenitor cells, which represent a significant population of the cells infected by ZIKV. Hence, the selective substrate repertoire may be affected by the localization of the protease within infected cells. Throughout the human proteome, protease recognition motifs are abundantly present. However, only a limited number of proteins undergo cleavage during infection, suggesting that cleavage of host protein may also be determined by the accessibility of that protein.

## 5 Discussion

The growing interest in DENV and ZIKV NS3 proteases extends beyond their roles in viral polyprotein processing. NS2B3 interacts with and cleaves numerous host proteins, thereby reprogramming cellular pathways to support viral replication, immune evasion, and pathogenesis (Khan et al., 2024). Proteolytic targeting of host substrates enables timely remodeling of cellular machinery, facilitating stress adaptation and creating a favorable environment for viral proliferation (Chen et al., 2017; He et al., 2025).

NS3 exhibits defined substrate specificity by recognizing dibasic residues (Lys/Arg) at the P2 and P1 positions with small residues at P1′. A compatible cleavage motif is preferentially required for NS2B3-mediated cleavage of host proteins. For example, NS2B3 efficiently cleaved mammalian SQSTM1 but not avian SQSTM1 from birds because the avian protein contains Gln rather than Lys/Arg at the P2 position (Zhou et al., 2024). However, substrate recognition is not determined solely by primary sequence, as highlighted by the cleavage of eIF4G1, which also depends on structural accessibility and protein context (Hill et al., 2018). Through selective proteolysis, orthoflaviviruses reprogram host translation and nuclear transport pathways to enhance viral protein synthesis while suppressing antiviral responses (De Jesús-González et al., 2020; Liu et al., 2021).

NS3-mediated cleavage of host factors may contribute to disease severity. In DENV infection, loss of Nrf2 signaling disrupts redox homeostasis, leading to excessive accumulation of reactive oxygen species, thereby enhancing viral replication and driving inflammatory and apoptotic gene expression (Ferrari et al., 2020). Stabilizing the balance of mitochondrial function with controlled production of ROS is important for pDCs to optimally produce IFN I. Disruption of redox equilibrium may negatively impact the capacity to mount antiviral responses and may lead to inflammatory tissue damage. Because NS2B3 in DENV and ZIKV perturbs the redox balance and regulates mitochondrial function, elevated ROS and coupled with reduced Nrf2 levels may be the result of NS2B3-mediated Nrf2 cleavage. pDCs are also important for the regulation of DENV infection, producing IFN-α and exhibiting TNF-related apoptosis inducing ligand (TRAIL)-mediated antiviral activity (Gandini et al., 2013). In DENV infection, TRAIL and IFN-α production by TRAIL-expressing pDCs correlate with positive outcomes in DENV infection. Unlike many other substrates highlighted in this review, Nrf2 suppression is linked predominantly to indirect lysosomal degradation rather than direct NS2B3 proteolytic cleavage, and no definitive viral protease cleavage site has been identified for Nrf2.

Other studies have shown NS2B3-mediated cleavage of MFN1 and MFN2 disrupts mitochondrial dynamics, and GrpEL1 may be a target of DENV NS2B3 and a contributor to mitochondrial dysfunction, as well as increased inflammatory responses and stress signaling (Yu et al., 2015; Gandikota et al., 2020). Dengue patients exhibit elevated serum λ FLCs that correlate with disease severity, whereas NS3-mediated antibody degradation reduces neutralizing capacity and weakens humoral immunity (Wang et al., 2023). Reduced levels of EDRF1 and GrpEL1 in dengue patients may provide a mechanistic explanation for dengue-associated platelet depletion (Gandikota et al., 2020; Gandhi et al., 2023). By disrupting mtHsp70-GrpEL1 chaperone function, DENV protease compromises mitochondrial homeostasis, contributing to metabolic dysfunction and disease severity, and highlighting NS3 as a central virulence factor and potential therapeutic target (Gandikota et al., 2020; Gandhi et al., 2023). Although both viruses use the protease to regulate translation and cytoskeletal organization (Hill et al., 2018; Shah et al., 2018; Li et al., 2019a), ZIKV NS3 more prominently targets proteins involved in autophagy, cell cycle, cytoskeleton, and development, which are potentially associated with neurodevelopmental defects (Li et al., 2019a). However, for several nucleoporins, the precise NS2B3 cleavage sites have not yet been fully identified or biochemically mapped. Accordingly, these proteins should currently be regarded as protease-sensitive host factors associated with NS2B3-mediated NPC disruption, rather than as fully validated direct cleavage substrates.

In addition to directly cleaving key components of the innate immune signaling cascade, NS3 is itself counteracted by host antiviral mechanisms. Interferon-stimulated genes, including viperin, Tripartite motif-containing proteins 22 (TRIM22), TRIM38, and C19orf66, inhibit ZIKV infection by binding NS3 and promoting its proteasomal or lysosomal degradation (Panayiotou et al., 2018; Wu et al., 2020; Zu et al., 2022; He et al., 2025). Moreover, S100A6 is a calcium-binding protein involved in cellular processes, including activity against orthoflavivirus. ZIKV infection promotes cellular expression of host S100A6, which binds to ZIKV NS3 and causes its lysosomal degradation (Peng et al., 2025). This dynamic interplay between viral protease activity and host countermeasures represents an important regulatory axis that could be exploited to develop antiviral therapeutics against DENV and ZIKV.
